# Matrix Metalloproteinases Inhibitors in Cancer Treatment: An Updated Review (2013–2023)

**DOI:** 10.3390/molecules28145567

**Published:** 2023-07-21

**Authors:** Shriefa Almutairi, Hanin Moh’d Kalloush, Nour A. Manoon, Sanaa K. Bardaweel

**Affiliations:** 1 Department of Pharmaceutical Sciences, School of Pharmacy, University of Jordan, Amman 11942, Jordan; shriefaalmutairi@gmail.com (S.A.); haneen.kalloush@zuj.edu.jo (H.M.K.); nourmanoon@gmail.com (N.A.M.); 2 Department of Pharmacy, Al-Zaytoonah University of Jordan, Amman 11733, Jordan

**Keywords:** matrix metalloproteinases (MMPs), inhibitors, anticancer activity, extra-cellular matrix remodeling

## Abstract

Matrix metalloproteinases (MMPs) are identifiable members of proteolytic enzymes that can degrade a wide range of proteins in the extracellular matrix (ECM). MMPs can be categorized into six groups based on their substrate specificity and structural differences: collagenases, gelatinases, stromelysins, matrilysins, metalloelastase, and membrane-type MMPs. MMPs have been linked to a wide variety of biological processes, such as cell transformation and carcinogenesis. Over time, MMPs have been evaluated for their role in cancer progression, migration, and metastasis. Accordingly, various MMPs have become attractive therapeutic targets for anticancer drug development. The first generations of broad-spectrum MMP inhibitors displayed effective inhibitory activities but failed in clinical trials due to poor selectivity. Thanks to the evolution of X-ray crystallography, NMR analysis, and homology modeling studies, it has been possible to characterize the active sites of various MMPs and, consequently, to develop more selective, second-generation MMP inhibitors. In this review, we summarize the computational and synthesis approaches used in the development of MMP inhibitors and their evaluation as potential anticancer agents.

## 1. Introduction

Matrix metalloproteinases (MMPs) are a member of the enzyme group that is capable of protein degradation [1]. MMPs are recognized as metalloproteinases because they require either zinc and calcium to perform their functions [2]. The catalytic activity of MMPs was first identified in 1962 as collagen proteolytic enzymes [3]. Since that moment, the research field of MMPs has undergone extensive advancement. In humans, 23 structurally related MMPs are known. Members of this family belong to the metzincin superfamily of proteases that can mainly degrade or cleave many components of the extra-cellular matrix (ECM) during tissue remodeling [4]. The ECM is an intricate network of macromolecules that provides biochemical and structural assistance to neighboring cells by regulating physiological activities such as cell anchorage, migration, proliferation, differentiation, and metabolism [5,6]. ECM degradation is also associated with several pathological events, including cancer [7,8]. Proteoglycans, collagens, fibronectin, elastin, and laminins are examples of the major glycoproteins of ECM [9].

MMPs can be categorized into six groups based on substrate specificity and structure differences (Table 1): collagenases, gelatinases, stromelysins, matrilysins, metalloelastase, and membrane-type MMPs [10]. The MMPs’ substrates are different, in that some are considered constituents of the ECM, whereas others are constituents of the basement membrane [10,11]. Even though each MMP has its own set of substrates, substantial overlap in substrate specificities between MMPs is common (Table 1) [5]. For example, MMP-1, which belongs to the collagenase subcategory, is responsible for cleaving native fibrillar collagens and more efficiently cleaving collagen of type III [11], but it can degrade laminin as well, which is mainly cleaved by MMP-3 and MMP-7 [10]. In addition, the favored substrate of MMP-2 is gelatin, which can also be degraded by MMP-3, -7, -12, or -14 [10]. Moreover, MMP-12, -2, and -9 overlap in their substrate specificity and elastin degradation ability [12].

MMPs are secreted by multiple connective tissues and pro-inflammatory cells such as fibroblasts, osteoblasts, endothelial cells, and macrophages [13]. Major sources of MMPs are dermal fibroblasts and leukocytes, especially for MMP-2, whereas MMP-1, -3, and -14 are highly expressed by platelets [13,14]. Also, MMP-1, -2, -3, and -7 are localized in endothelial cells and vascular smooth muscle cells (VSMCs), and MMP-12 is localized in fibroblasts of the human great saphenous vein, in addition to VSMCs and inflammatory macrophages [13,15].

MMPs are mostly expressed in an inactive form (pro-enzyme), which is subsequently activated by other proteolytic enzymes. MMPs are naturally regulated at any of the following three known levels: the transcription level (mRNA), the pro-enzyme activation level, and inhibition of the active forms by various tissue inhibitors of MMPs (TIMPs). In normal physiological conditions, MMPs form a 1:1 complex with endogenous tissue inhibitors of MMPs (TIMPs), which selectively modulate MMPs’ function [16]. On the other hand, under pathological conditions, this equilibrium is shifted towards increased MMP activity, leading to abnormal ECM metabolism and disease conditions [5].

**Figure 1:**
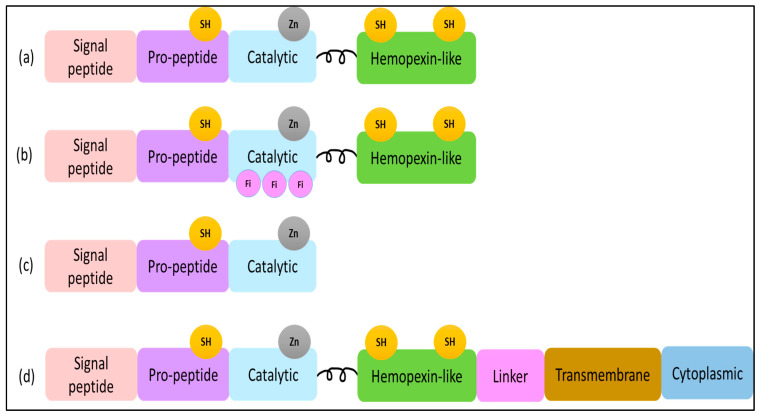
(**a**) MMP-1, -3, -12 structure domains. The minimal domains of MMPs are an amino-terminal **signal peptide** (SP), a **pro-peptide**, and a **catalytic** domain. The **SP** domain shows a role in guiding MMP-12 to the endoplasmic reticulum, and the **pro-protein** domain contains the thiol (SH) group that maintains MMP as inactive zymogens by interacting with zinc. (**b**) MMP-2 structure domains contain three fibronectins within the catalytic domain. (**c**) MMP-7 structure domains. (**d**) MMP-14 structure domains contain a linker, cytoplasmic domain, and transmembrane domain.

Despite the functional differences between MMPs, they are highly comparable in their structural domain [17]. The basic MMP structure consists of four main domains: an N-terminal pro-peptide domain, a catalytic domain, a linker region, and a C-terminal hemopexin-like domain (Figure 1) [18,19]. An exception to this highly conserved structure is the MMP-7 domain structure, which does not include the hinge region or hemopexin domain [10]. The pro-peptide domain is composed of around 80 amino acids in length and contains a cysteine switch motif that is responsible for the maintenance of enzyme latency. Meanwhile, the hemopexin-like domain has a C-terminal that is composed of approximately 200 amino acids and is connected to the catalytic region by a flexible proline-rich hinge region. The structure of the hemopexin-like domain has a fundamental role in several activities involving substrate specificity and activation, inhibition, anchoring, and dimerization [20,21].

The catalytic domain of MMPs is composed of around 170 amino acids and includes a zinc-binding site and methionine residue or “Met-turn”, which chemically supports zinc-binding activity, as well as a loop called a Ω-loop that surrounds an S1′ pocket region [19,22,23]. The protease’s catalytic part is compromised of three α-helices as well as a profoundly twisted five-stranded β-sheet. Three His residues, His199, His203, and His209, and a molecule of water in the active site cleft bind zinc in the amino acid sequence HELGHXXGXXH [24]. The active site of MMPs involves two discrete regions: a groove in the protein surface and an S1′ pocket [17]. The active site is known to be highly conserved among MMPs except for the S1′ pocket region, which varies slightly among MMPs in sequence, shape, and depth [17,25]. The S1′ pocket is accessed via a tunnel formed by different amino acids and is bounded by the Ω-loop, which is also diverse among MMPs in length, flexibility, and amino acid residues [17]. An additional feature of the S1′ pocket is the diversity of certain residues in the pocket. For example, Arg 218 in MMP-1 is replaced with Leu in MMP-2, -3, -12, and -14 and with Tyr in MMP-7 [17]. The S1′ pockets’ differences among MMPs have been relied upon to determine the selectivity of inhibitors.

As agents capable of remodeling ECM, MMPs have been linked to a wide variety of biological processes, such as cell transformation and carcinogenesis. It is now well-known that each MMP has a different role in a specific disease, and the role may change from unfavorable to favorable during the progression of disease stages. Interestingly, their role in the development of cancer may include various mechanisms. Originally, MMPs were thought to only associate with metastasis facilitation by ECM remodeling, thereby allowing for the invasion of tumor cells into blood and lymphatic vessels [18]. However, it is now confirmed that they support most tumorigenic processes, including proliferation, invasion, angiogenesis, and metastasis [10]. Furthermore, MMPs were reported to be overexpressed in various types of solid tumors, such as breast, prostate, liver, and lung cancers [18]. Likewise, several reports have shown the apparent expression of MMPs in non-solid tumors [18]. Nonetheless, MMPs’ expression and their functional significance in solid and non-solid tumors are yet to be fully clarified [26].

Considering their important roles in virtually all major stages of tumor progression, various MMPs have become attractive targets for diagnosis and therapeutics. The first generation of MMP inhibitors (MMPIs) was peptide-based. It was designed by employing MMP substrates as a pharmacophore model [5]. However, due to their molecular flexibility and poor bioavailability, the potency of these small peptides as drugs was substantially limited. Modifications of the natural peptide resulted in an ideal substitute termed “peptidomimetics” [27], which was further modified to hydroxamate-based peptidomimetics [5]. These compounds inhibit MMPs by coordinating with the catalytic Zn ^2+^ of MMPs via the zinc-binding group (ZBG). Batimastat and Marimastat represent pioneering hydroxamate-based MMPIs, displaying excellent anticancer activity in preclinical studies [5,28]. However, the results of clinical trials were not adequate. Subsequently, a series of hydroxamate derivatives was developed by applying structural studies such as X-ray crystallography and computer simulation analysis, which aid in improving their selectivity as MMPIs [5].

The second generation of MMPIs has different ZBG within both peptidomimetic and non-peptidomimetic motifs and include thiol-based, carboxylic-acid-based, pyrimidine-based, and hydroxypyrone-based MMPIs [5]. Although these inhibitors have considerable in vitro antitumor activity, early clinical trials demonstrated that such compounds have low oral bioavailability in addition to safety concerns [5]. Provided that these small molecular drugs cannot target specific active sites, the failure of their clinical trials was foreseeable.

The principal idea of MMPI design works via targeting the Zn^2+^ of the enzyme active site that is highly conserved and shares common features among most MMPs [17]. Accordingly, the non-specificity of MMPIs has been a fundamental reason for the failure of their clinical use [17]. With a further understanding of specific MMPs’ function and role in different tumors, substantial work has been put into inhibiting MMPs’ activity more selectively by targeting the catalytic site in addition to the molecular structures surrounding it [17].

Thanks to the evolution of X-ray crystallography, NMR analysis, and homology modeling studies, it has been possible to distinguish the active site of various MMPs and, consequently, to develop more selective, second-generation MMP inhibitors [11]. MMP inhibitors, often designed to address and accommodate the S1′ pocket flexibility [29], have the required selectivity and lack high toxicity [30].

Thus, a series of MMPIs with more selectivity has been generated [29,31]. Even with the accessibility of structural information, poor selectivity remains a challenge for the accomplishment of MMP inhibitors in clinical trials. Likewise, the intrinsic and ligand-induced flexibility of the active site makes its analysis more challenging. The present review discusses the recent development of MMP inhibitors and their evaluation as potential anticancer agents, particularly MMP-1, -2, -3, -7, -12, and -14, comprising one MMP from each subfamily. Databases were searched for the MMP inhibitors that were designed and synthesized in the period between 2013 and 2023.

## 2. Matrix Metalloprotease-1 (MMP-1)

### 2.1. Function and Localization

MMP-1 is an identifiable member of the MMP family. MMP-1 is responsible for breaking down interstitial collagen (types I, II, III, VII, and X); hence, it is also referred to as collagenase 1 [10,32]. In addition, it breaks down other substrates, such as gelatin, laminin, complement (C1), insulin-growth-factor-binding proteins, interleukin (IL-1), and tumor necrosis factor (TNF) [33,34,35,36]. MMP-1 also activates other MMPs, including MMP-9 and MMP-2 [37]. It plays a vital role in various biological processes, including angiogenesis, embryogenesis, morphogenesis, and the repair of wounds [38].

The gene encoding MMP-1 is localized to chromosome 11q22.3 [39]. MMP-1 is produced and released as an inactive proenzyme with 469 amino acids and a molecular weight of approximately 55 kilodaltons [40]. MMP-1 is released by various cell types and tissues, including endothelium, macrophages, fibroblasts, and platelets [40,41,42]. Furthermore, MMP-1 is found intracellularly, and evidence suggests that it might mediate signaling events that determine the cell’s function and phenotype [43]. MMP-1 has also been shown to be intracellular in several cells, including the vascular endothelium [44].

### 2.2. Role in Cancer

MMP-1 was linked to several diseases, including lung emphysema, arthritis, and especially cancer [3,4,5]. Recent studies suggest that MMP-1 promotes cancer cell invasion and migration and significantly correlates with malignancies’ unfavorable outcomes [45,46]. Increased MMP-1 expression has been observed in oral, bladder, gastric, and breast cancers and was linked to a poor prognosis for these diseases [47,48,49,50]. Research findings suggest that MMP-1 encourages the proliferation and invasion of breast cancer cells by cleaving the same Arg-Ser bond as thrombin do [51]. This eventually leads to activating the protease-activated receptor (PAR), which assists in the proliferation and invasion of breast cancer cells [51]. Moreover, Wang et al. found that MMP-1 has been linked to the promotion of malignant behavior in colorectal cancer via epithelial–mesenchymal transition EMT and the Akt signaling pathway [52]. Another study showed that increased MMP-1 and vascular endothelial growth factor-C (VEGF-C) expression was associated with an advanced tumor stage and a poor prognosis in patients with esophageal squamous cell carcinoma [53]. In addition, overexpression of MMP-1 was associated with the invasiveness of primary nodular melanoma [54]. Therefore, these findings indicate that a drug modifying MMP expression or activity might be used in cancer treatment.

### 2.3. Structure of the Catalytic Domain

The primary structural difference between MMP-1 and other MMP enzymes is the size and shape of S1’s pocket. The S1′ pocket of MMP-1 is short and narrow compared to other enzymes in the MMPs family [17]. MMP-1 inhibitors were developed by taking advantage of the presence of a metal ion inside the binding pocket, aside from the unique characteristics of the S1′ pocket [17,38]. Another study conducted by Gimeno et al. suggested that some inhibitors occupy the S1’ pocket interacting with the Ω-loop by protein–ligand docking of MMP-1 inhibitors of various sizes [17].

### 2.4. MMP-1 Inhibitors

Several synthetic chemical inhibitors have been created utilizing combinatorial chemistry and structure-based design [38]. The earliest designed compounds were broad-spectrum inhibitors targeting a wide range of MMPs. Thus, advanced clinical trials with them were unsuccessful due to their limited oral bioavailability, diminished in vivo efficacy, and adverse musculoskeletal effects [38]. Musculoskeletal syndrome (MSS) was the most prominent side effect with an unknown exact origin [55]. However, MMP-1 inhibition was first thought to be a contributing factor. As a result, MMP inhibitors that spare MMP-1 have gained more attention [55].

Over the past decade, new MMP-1 inhibitors have been developed using structure-based design techniques such as methyl rosmarinate derivatives. A series of methyl rosmarinate derivatives was assessed as selective MMP-1 inhibitors, and compound **1** was the most potent candidate with an IC_50_ of 0.4 µM (Table 2) [56]. Moreover, Umedera et al. have investigated new MMP-1 inhibitors using a structure–activity relationship (SAR) transfer method based on kinesin-like protein 11 (KIF11) analogs [57]. Among the candidate compounds generated, compound **2** was the most selective inhibitor with an IC_50_ of 0.034 µM (Table 2) [57]. Additionally, a series of thiazole derivatives was examined as MMP anti-neoplastic agents, and compound **3** was the most promising agent. It exhibited multiple MMP inhibition (MMP-1, MMP-8, and MMP-9). with an MMP-1 inhibitory activity of 10% ((at 1.3 µM concentration) (Table 2)). Moreover, it was the most effective and selective anticancer agent against the MCF-7 cell line, with an IC_50_ of 20 µg/mL [58]. An integrated structure-based approach was also used to design a new potent inhibitor utilizing an arylsulfonamide scaffold. Nevertheless, all the designed compounds were non-selective, and the most potent MMP-1 inhibitor was compound **4**, which exhibited a ki value of 77 nM (Table 2) [59]. A SAR matrix approach was utilized by Asawa et al. to develop new inhibitory compounds against MMP-1. They also carried out an experimental validation for the predicted inhibitory compounds, and the most potent inhibitor was compound **5,** displaying an IC_50_ of 0.18 µM (Table 2) [60].

## 3. Matrix Metalloprotease-2 (MMP-2)

### 3.1. Function and Localization

The 72KDa MMP-2 enzyme is quite remarkable because it can act on a wide variety of substances including gelatin, laminin, fibronectin, elastin, and collagen IV [61]. Because of this, it was previously classified as part of the collagenase IV subfamily [61]. Once MMP-5 was discovered, scientists decided to remove its numbering, because they found it was identical to MMP-2 in terms of its genetic composition [62,63].

MMP-2 plays a major role in the proteolytic decomposition of ECM components [64] and basement membrane. Therefore, MMP-2 inhibition tends to be a target for tissue repair and cancer metastasis [65,66,67,68]. The gene encoded MMP-2, which is localized on chromosome 16q13 [69].

### 3.2. Role in Cancer

Tumor cells can produce MMP-2 or stimulate its production in surrounding cells. This production creates an environment that helps tumor cells grow and spread [70]. Also, MMP-2’s ability to degrade the ECM components enhances cancer cells’ migration through tissues [70]. Moreover, MMP-2 can affect signaling pathways in cancer cells by promoting tumor cell growth, enhancing resistance to apoptosis, and improving angiogenesis [71]. MMP-2 is overexpressed in many types of cancer, including bladder [72], breast [73], bronchopulmonary [74], cervical [75], colon, glioma [76], laryngeal [77], lung, melanoma, myeloma, esophagus, ovary [78], pancreas, prostate, skin, and stomach cancer [64,65,71].

### 3.3. Structure of the Catalytic Domain

MMP-2 stands out from other MMPs in that it has three head-to-tail repeats within the catalytic domain [71] and contiguous fibronectin type-II-like (FN-II) motifs [64,79]. Those motifs are bound to the extra 175 amino acid residues [64] and are involved in destroying and remodeling ECM components [64,79]. Scientists believe that targeting both the active site and the collagen-binding FN-II domains will show promising effects in inhibiting the MMP-2 role in cancer [80].

Studies have revealed the presence of two hydrophobic domains designated as the S1′ and S1 pockets, in addition to the zinc^2+^ ion pocket [64]. Notably, the S1′ pocket is considered the predominant pocket of MMP-2 and is characterized by its relatively narrow and deep shape compared to other MMP subtypes [17]. Current research efforts have led to the identification of various structural features that are common to most MMP-2 inhibitors, including a zinc-binding group, a non-zinc-binding group, a hydrogen-bond-forming functional group, and one or more hydrophobic side chains that interact with the S1′ and S1 pockets [64]. Moreover, the hemopexin-like domain (HDM) of MMP-2 has a four-bladed propeller around a central cavity occupied by a Ca^2+^ ion [79]. HDM is linked to the catalytic domain through a flexible hinge region. According to a study [81], HDM plays a role in allowing MMP-2 to interact with integrin α_V_β_3_, a protein, in melanoma cells and endothelial cells, even without the presence of the Arg-Gly-Asp (RGD) motif that is typically involved in such interactions [82].

MMP-2 is stimulated as a reaction to tissue damage, wound healing, and immune response [83,84]. Its production is regulated by numerous factors, such as growth factors, cytokines, and hormones [85]. Moreover, it can be activated in response to different physiological and pathological conditions [33]. However, excessive activation of MMP-2 has been linked to various health problems such as cancer and arthritis, whereby it promotes the growth and spread of tumors and contributes to tissue destruction and joint damage [86].

### 3.4. MMP-2 Inhibitors

In recent years, computational chemistry techniques along with machine-learning and artificial intelligence approaches [87] have been extensively explored for designing effective MMP-2 inhibitors [65,66,67,88,89,90,91] with particular binding patterns. This has enabled the development of numerous small-molecule MMP-2 inhibitors that have shown promising preclinical results [25,68,88,92,93,94,95].

Different synthesis techniques have executed a major impact on the development of MMP-2 inhibitors through the modification and optimization of lead compounds [65,66,67,88,96]. The synergy between computational chemistry and synthesis techniques has allowed researchers in the last decade to develop new small-molecule MMP-2 inhibitors, as shown in Table 3, specifically designed to target MMP-2 [25,29,65,66,88,96]. These inhibitors have been demonstrated to exhibit antitumor effects in both in vitro and in vivo [65,66,88,96].

About 72 molecules with different scaffolds were virtually screened (compound **6**) using Regression-dependent quantitative structure–activity relationship (QSAR) strategies. The aim was to determine the structural features that are needed to find a suitable MMP-2 inhibitor [89]. Turra et al. utilized 4D-QSAR and pharmacophore modeling to study a group of 40 different chemical β-N-biaryl ether sulfonamide hydroxamate derivatives (compound **7**). Their approach was used to predict the inhibitory activity of the compounds against MMP-2 [97].

A scaffold modification technique was used by Qiu et al. to design and synthesize a new set of MMP-2 inhibitors. The synthesized compounds were evaluated against several cancer cell lines, including A549, HepG2, MCF-7, and Hela cells. Among them, compound **8** showed the highest potency against MMP-2. Also, compound **8** shows almost no cytotoxicity against 293T kidney cells [66].

A series of sulfonamide’s derivatives, dihydropyrazole sulfonamide derivatives that contain 2- hydroxy phenyl moiety (compound **9**), were synthesized by Wang et al. as MMP-2 inhibitors with anti-cancer activity. Bioactive assays on four cancer cell lines, A549, MCF-7, Hela, and HepG2 cells, were conducted, revealing a potential antagonistic effect on MMP-2. Also, molecular docking SARs were performed on the derivatives and demonstrated that compound **10** exhibits the most potent inhibition potentials [96].

The same team reported the synthesis of a new series of sulfonamide derivatives (compound **11**) that contain dihydropyrazole moieties [98]. The reported compounds were found to have a dual effect on MMP-2 and -9. After testing on the same cell lines, the most active compound was compound **11** compared to the control positive compound [98].

Halder et al. reported the design and the biological evaluation of compound **12**, which acts on dual targets and shows selective activity toward MMP-2/HDAC-8 among their subtypes over A549 cell line lung carcinoma [99]. In addition to this, Ammazzalors et al. reported the synthesis as well as the molecular and biological evaluation of simple derivatives of hydroxyquinoline and hydroxynaphtyridine (compound **13**) to be selective inhibitors toward MMP-2 and -13. These compounds are reported to have much fewer side effects because of the zinc-binding group in their structures [91]. Another reported compound (compound **14**), which contains salicylaldehyde, was synthesized. This compound was found to exhibit dual inhibitory activity against MMP-2 and -8 [100]. The synthesis was achieved by cross-condensation reactions, and the biological evaluation was performed against MCF-7, Hela, and HepG2 cell lines [100].

Another dual inhibitory effect of the gelatinase enzymes was reported by Chen. et al., who designed and synthesized 8-hydroxyquinoline derivatives (compound **15**) [65]. The most active compounds showed a good inhibitory effect toward MMP-2 and -9 and possessed potent anti-proliferative activity against different cancer cell lines, including MCF-7, PC-3, HL-60, K562, KG1, and A549 [65]. In addition to this, the compounds also had anti-invasive and anti-angiogenesis activity on A549 lung cancer cells [65].

Compound **16** was designed and synthesized with microwave assistance, mimicking the non-hydroxamate inhibitors [67]. The synthesized compounds were reported by Albelwi et al. to possess antagonistic activity toward MMP-2/9 [67].

The microwave-assisted technique was also used in triazoles as reported by Aouad et al. via various sulfonamide bridges in compound **17** [101]. The synthesized compounds were reported to have dual action on other targets including MMP-2 [101].

According to Kreituss et al., a series of (aryl triazolyl) methyl aziridines compounds was synthesized and evaluated for their selective inhibition of MMP-2 [102]. Compound **18** is made up of a hydrophilic aziridine, a lipophilic part, and a triazole fragment that connects the two [102]. Among all the synthesized inhibitors that were tested against melanoma and PT-67 fibroblast cell lines, compounds **19** and **20** showed the best inhibition of MMP-2, with a concentration of 20 and 10 µM, respectively [102].

Laghezza et al. reported a new non-zinc-binding MMP-2 inhibitor [103]. This was achieved by checking previously known compounds using virtual screening along with SAR for benzimidazole structure, resulting in compound **21**. Simple molecules were synthesized, and their activity was measured [103]. Molecular dynamics were checked to confirm the design of more selective MMP-2 inhibitors [103].

Researchers have attempted to develop gelatinase inhibitors that can cross the blood–brain barrier. Iproteos company researchers in Spain developed new gelatinase inhibitors, compounds **22** and **23**, that were capable of inhibiting MMP-2 and MMP-9 with high potency and selectivity among different MMPs [11,104].

**Table 3:** Chemical structures and potencies of synthetic MMP-2 inhibitors.

Compound	Potency	Chemical Structure	Reference
**6**	-	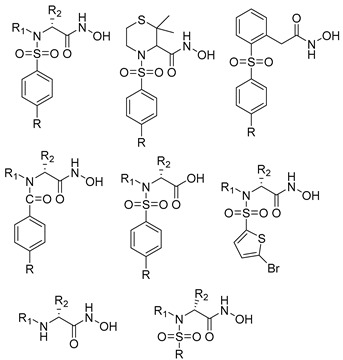	[89]
**7**	*p*IC_50_ (−logIC_50_) = 8.60“IC_50,_ nM”	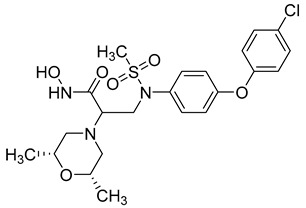	[97]
**8**	IC_50_ = 0.38 µM	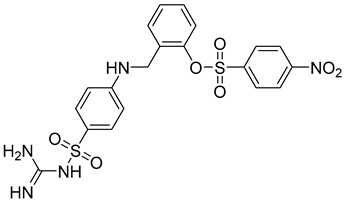	[66]
**9**	IC_50_ < 5 µM	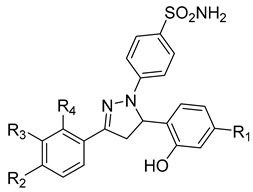	[96]
**10**	IC_50_ = 0.33 µM	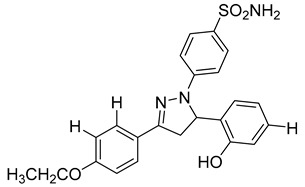	[96]
**11**	IC_50_ = 0.21 µM	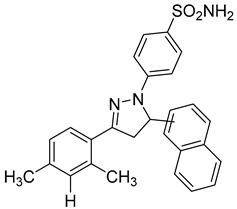	[98]
**12**	IC_50_ = 6.40 µM	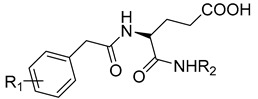 R1 = 4Br/R2= CH_2_C_6_H_4_ 4-Nitrobenzyl	[99]
**13**	IC_50_ = 7.4 ± 0.8 µM	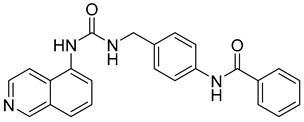	[91]
**14**	IC_50_ = 2.80 µM	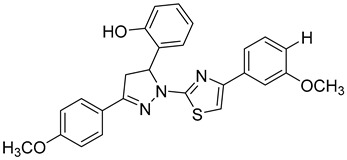	[100]
**15**	IC_50_ = 0.70 ± 0.02 µM	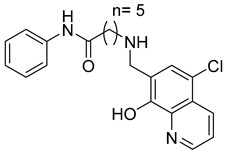	[65]
**16**	IC_50_ = 0.376 µM	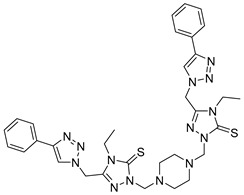	[67]
**17**	IC_50_ = 5.6 nM	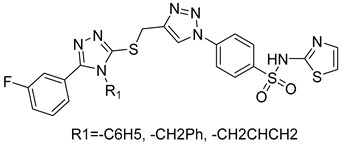	[101]
**18**	-	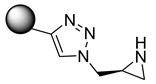	[102]
**19**	Percent of inhibition = 73.3%	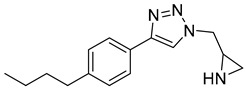	[102]
**20**	Percent of inhibition = 75.2%	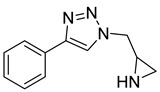	[102]
**21**	IC_50_ = 31 ± 5 µM	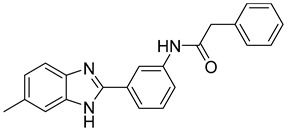	[103]
**22**	IC_50_ = 21 nM	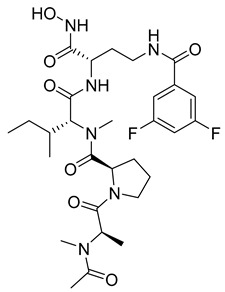	[104]
**23**	IC_50_ < 1nM	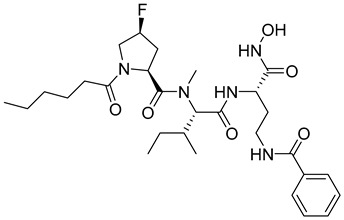	[104]

## 4. Matrix Metalloprotease-3 (MMP-3)

### 4.1. Function and Localization

MMP-3 is a recognized endopeptidase that belongs to the stromelysins subfamily [105]. The MMP-3 protein comprises 475–478 amino acids in mammals with an approximated molecular weight of 57 kilodaltons [40,106]. The MMP-3 gene is localized in chromosome 11q22.3 [107]. Regarding MMP-3 subcellular localization, MMP-3 has been found in the nucleus and might be involved in the transcriptional or apoptosis processes in some cells, such as hepatocellular carcinoma, chondrocytes, and myofibroblast [108,109,110]. Furthermore, it is an exocrine protein typically secreted via the exocytosis process and extracellular vesicles [111]. Numerous cell types produce MMP-3, including chondrocytes, endothelial cells, macrophages, and fibroblasts [112,113,114].

MMP-3 is termed stromelysin-1 owing to its hydrolytic ability and stromal origin [105]. MMP-3 has distinct structural features that facilitate its role in hydrolyzing numerous extracellular matrix proteins (EMP), including collagen types II and III, laminin, fibronectin, and proteoglycans [111]. Further, it can dissolve adherent junctions through its activity on a cell-surface protein called E-cadherin. MMP-3 also activates tissue-remodeling MMPs, namely, pro-MMP-1 and pro-MMP-9 [115,116]. Additionally, MMP-3 is an efficient plasminogen activator via its interaction with tPA (tissue-type plasminogen activator) [117]. The broad substrate specificity of MMP-3 facilitates its contribution to a range of pathological disorders, including malignant, neurodegenerative (e.g., Alzheimer’s), and inflammatory joint diseases (e.g., rheumatoid arthritis) [118,119].

### 4.2. Role in Cancer

Concerning its role in malignancy, studies have suggested that MMP-3 has both tumor-promoting and tumor-inhibiting properties, depending on the substrates it interacts with [120]. For instance, MMP-3 catalyzes the formation of angiogenesis-inhibiting factors through the breakdown of plasminogen and type VIII collagen [121,122]. These factors will restrict tumor progression; MMP-3 demonstrates tumor suppression properties in such a case. While modulating growth factors (e.g., transforming growth factor), MMP-3 stimulates the proliferation of cancer cells and tumor progression [120,123]. Nevertheless, MMP-3 is more commonly involved in promoting tumor development than suppressing it [120].

MMP-3 is considered a prognostic factor in several types of cancers. Camacho et al. [117] reported an increased expression of MMP-3 in breast cancer tissue compared to normal breast tissue, which might be associated with breast cancer development. Interestingly, a study by Cai et al. found an upregulated expression of MMP-3 in oral squamous cell carcinoma, the most common oral cancer [124]. Moreover, recent research implies that MMP-3 has a role in prostate cancer progression to bone metastasis [125]. Chen et al. have also demonstrated the crucial role of ubiquitin-specific peptidase 15 and MMP3 in non-small-cell lung cancer development and prognosis [126]. Therefore, MMP-3 is a promising target for the generation of new antineoplastic drugs.

### 4.3. Structure of the Catalytic Domain

The hydrophobic S1′ pocket of MMP-3 has a tunnel-like shape, as revealed by X-ray crystallography [127]. Compared to other MMPs, this MMP3’s pocket is classified as deep and large, likely due to the length of the Ω-loop sequence that affects MMP-3 conformations [17]. Leu229’s side chain and the side chains of Leu226 and Thr227 in specific conformations restrict substrates’ entry into the bottom of the S1′ pocket of MMP-3 [17]. These unique characteristics of the S1′ pocket account for the distinct substrate specificity of this enzyme [37].

### 4.4. MMP-3 Inhibitors

Several anticancer agents have been designed to target the catalytic hydrophobic pocket of MMP-3. One of the earliest MMP-3 inhibitors features the hydroxamate-based zinc-binding scaffold **24** with a Ki of 43 nM (Table 4). Unfortunately, this scaffold failed in clinical trials due to insufficient oral bioavailability and cartilage matrix adverse effects [38]. However, such inhibitors were utilized as a model for developing selective second-generation hydroxamate-based inhibitors [38]. On the other hand, Pavlovsky et al. have designed MMP-3 inhibitors with a carboxylic acid moiety that displays a low Ki value of 19 nM for the S enantiomer **25**. Another study was conducted by Brown et al., which disclosed a potent thiirene-containing covalent-bond-forming inhibitor **26** with a Ki of 15 nM [38]. A series of 2-phthalimidinoglutaric-acid-based analogs were designed in silico as MMPs inhibitors. Among the designed inhibitors, compound **27** demonstrated auspicious predicted activity against MMP-3, with an approximated IC_50_ of 1 nM [128]. Recently, research findings have shed light on a series of sulfonamide-containing dehydroabietic-acid (DHAA)-based derivatives as MMPs inhibitors. Surprisingly, compound **28** exhibited selectivity towards MMP-3, with an IC_50_ of 0.4 µM [129].

## 5. Matrix Metalloprotease-7 (MMP-7)

### 5.1. Function and Localization

MMP-7 (matrilysin-1) is a small secreted proteolytic enzyme that functions as zinc and calcium endopeptidase [130]. The gene encoding MMP-7 belongs to the gene cluster on chromosome 11 at q21 and q22 [131,132]. The complete coding sequence of MMP-7 is 1094 bp long and encodes 267 amino acids [133].

MMP-7 is produced as a pro-enzyme with a molecular weight of 28-kDa [134]. The full length of MMP-7 consists of two structural domains: the propeptide domain and the catalytic domain [135]. The activation process of pro-MMP-7 involves a proteolytic removal of the propeptide domain (9-kDa) that is responsible for the latency of the enzyme [136]. MMP-7 is the smallest member of the MMP family, because it lacks a further functional region called the hemopexin-like domain [137]. The hemopexin-like domain is responsible for substrate specificity [1,137]. Thus, the absence of this domain conforms with MMP-7 activities against a wide range of ECM components such as collagen (IV–X), fibronectin, laminin, and gelatin [138].

In addition, MMP-7 takes a part in the degradation of some non-ECM proteins such as pro-α-defensin, pro-tumor necrosis factor (TNF)-α, and E-cadherin [21]. In addition, MMP-7 also activates other MMPs, namely, pro-MMP-1, -2, -9, and -8 [139]. Under normal circumstances, MMP-7 is secreted by epithelial cells, normal endometrial, bronchial, ductal, skin glandular urogenital, and gastrointestinal tissues, and some macrophages [132,134,135]. Thus, it plays a critical role in the management of several processes, including inflammatory processes, aging, and bone growth as well as remodeling and signaling pathways that control cell growth, inflammation, and angiogenesis [132,140,141,142].

### 5.2. Role in Cancer

A substantial body of evidence indicates that overexpression of MMP-7 plays an integral role in tumor pathogenesis and progression [134,135,143,144]. Tumor pathogenesis is a sequential process implying cell growth, invasion, metastasis, and angiogenesis [134]. Abnormally high expression of the gene encoding MMP-7 can promote tumor progression by inhibiting cancer cells’ apoptosis [145], reducing cell adhesion [134,146], and inducing angiogenesis [147].

Like other MMPs, MMP7 promotes cancer invasion by the proteolytic degradation of ECM proteins [134,135]. Also, MMP-7 activation of pro-MMP-2 and -9 can facilitate tumor invasion. Additionally, MMP-7 promotes tumor invasion through its role in the regulation of non-ECM components. MMP-7 breaks down β4 integrin and E-cadherin, which function as positive regulators of cell adhesion [134,146]. Thus, the degradation of these components promotes cancer migration and invasion [134]. Additionally, MMP-7 has an anti-apoptotic effect by degrading insulin-like growth factor 3 (IGFBP-3). IGFBP-3 is a biologically active growth factor that has a role in promoting tumor cell proliferation [148]. Moreover, MMP-7 was confirmed to directly accelerate angiogenesis by activating the proliferation of vascular endothelial cells [149].

MMP-7 is abundantly expressed in many types of cancer tumors, including breast [150], colon [151], oesophageal [152], pancreatic [153], and lung cancer [143]. In addition, MMP-7 is associated with an aggressive cancer phenotype and poor prognosis in patients with gastric cancer [154]. MMP-7 is also linked to clinicopathological aspects such as tumor stage by predicting the histological grade of the tumor [155]. Also, MMP-7 can act as a potential molecular marker for cancer diagnosis [150].

### 5.3. Structure of the Catalytic Domain

The catalytic domains of MMPs share a subsequent similarity, where the percentage of similarity ranges between 33% and 86% [1]. The catalytic domain of MMP-7 has a ball-like structure with three α-helices, five β-sheets, and multiple loops [156]. It also contains two Zn^2+^ binding sites. The first binding site is a structural site with Zn^2+^ coordinated by three conserved histidine and aspartic acid molecules. The second binding site is a catalytic site where Zn^2+^ is coordinated by three conserved histidine and 1–2 solvent molecules [156].

In MMP-7, the residue equivalent to Leu218 of MMP-13 is Tyr215. With this particular residue, the S1′ pocket of MMP-7 is classified as shallow and small [29]. In MMP-7, Tyr215 adopts a conformation that prevents the binding between ligands and the S1′ pocket. Hence, this residue creates a steric hindrance for those ligands that interact with the Ω-loop and explains the selectivity of ligands that perform these types of interactions. This is illustrated by the docking on MMP-7 of several MMP inhibitors that show selectivity over MMP-7. Therefore, the size and shape of the S1′ pocket are crucial determining factors in the selectivity of many MMP inhibitors.

### 5.4. MMP-7 Inhibitors

MMP-7 has been identified as a validated drug target and anti-target for cancer therapy [157]. When downregulation of MMP-7 restores the normal state of the cell and tissue, it is considered a drug target. In cancer, such drugs result in cancer cell death or slow disease progression. On the other hand, downregulation of MMP-7 may result in clinically unacceptable side effects, the initiation of cancer, or deleterious alterations in disease progression. In this case, MMP-7 is considered a drug anti-target [157]. Hence, improving selective inhibitors for MMP-7 could induce the evolution of cancer management [158]. As explained earlier, S1′ is the key determinant for MMP-inhibitor selectivity. However, because both MMP-7 and MMP-1 share similar S1′ features, it remains an ongoing challenge to determine the selectivity of their inhibitors [29].

Li et al. have introduced a nitro group as a ZBG of MMP inhibitors [159]. They demonstrated that reasonable modification of the P3′ side chains of the nitro-based MMP inhibitors has enhanced the selectivity of inhibition for MMP-7 over MMP-1 [159]. A series of compounds were synthesized and evaluated as MMP inhibitors by molecular docking [159]. Five compounds presented the best positions based on the distance of their nitro group from the catalytic ZN^2+^ and the binding free energies [159]. However, one compound (compound **29**) demonstrated a higher selectivity of MMP-7 over MMP-1 [159]. The inhibitory constant (*Ki*) of compound **29** to MMP-7 was 3.7 µM [159]. The structure of the compound scaffold is shown in Table 5.

Fischer and Riedl aimed to improve the inhibition activity against MMP-7 by modifying a non-hydroxamate selective inhibitor previously identified as MMP-13 [158]. A series of modifications was accomplished, and the synthesized compounds were examined in vitro to predict their potency against MMP-7 [158]. Compound **30** was identified as the most potent inhibitor against MMP-7, with an IC-50 of 2.2 µM (Table 5). The authors noted that these modifications not only improve the selectivity against MMP-7 but also decrease the potency of the original compound toward MMP-13 [158].

## 6. Matrix Metalloprotease-12 (MMP-12)

### 6.1. Function and Localization

MMP-12 is an elastolytic protease that is primarily produced by inflammatory macrophages. Thus, it is also known as macrophage metalloelastase or macrophage elastase [15]. The significant role of MMP-12 in inflammation is demonstrated by its capability to degrade the basement membrane, which leads to macrophages’ penetration into injured tissue [160]. MMP-12 can degrade several extracellular matrix (ECM) structures, such as type IV collagen, fibronectin, fibrillin-1, laminin, vitronectin, chondroitin sulfate, and heparin sulfate proteoglycans. Furthermore, it can break down elastin, which is widely distributed in the lung [19,160].

MMP-12 is localized in the cytosol and nuclei of various cells [161]. The MMP-12 gene, like other MMPS genes, is positioned on human chromosome 11, at 11q22.3 [162]. MMP-12 is produced as a 54 kDa pro-form enzyme that goes through self-activation by autolytic processing, resulting in the generation of 45 kDa and 22 kDa active forms [19,160]. Alongside its autolytic processing activity, MMP-12 activates other MMPs such as pro-MMP-2 and pro-MMP-3, which, in turn, activate pro-MMP-1 and pro-MMP-9. This might be the reason behind MMP-12’s ability to amplify a cascade of proteolysis that leads to the degradation of a wide variety of ECM proteins.

The activity and expression levels of MMP-12 are controlled by multifaceted mechanisms involving different pathways at transcription, post-transcription, translation, and post-translation levels [18]. Of note is these mechanisms can impact the proteolytic and non-proteolytic functions of MMP-12 [18]. The transcriptional regulation of MMP-12 activity involves MMP-12 gene expression, transcript stability, promoter polymorphisms, and epigenetic alterations [18]. The post-transcriptional mechanism incorporates the regulatory effect of microRNAs (miRNAs) on the expression of one or multiple target genes. An example is the regulatory effect of miRNA-452 by targeting MMP-12 encoding [163,164]. Numerous studies have shown the importance of miRNAs in the development of cancer, specifically lung cancer [165,166,167,168,169].

Moreover, MMP-12 expression is upregulated at both mRNA and protein levels in visceral and subcutaneous white adipose tissue from obese mice and humans [170]. In addition, MMP-12 upregulation is subject to cellular differentiation status, which was proven by its non-existence in monocytes, the cells from which macrophages originate [19].

The location (compartmentalization) of the enzyme is another important factor regulating MMP-12 activity [171]. Localization stands for the congregation of MMPs around potential substrates or restricted availability of their endogenous inhibitors [171]. Secreted MMPs are found to bind to cell-surface membranes and therefore trigger intracellular cascade pathways [171]. Interestingly, extensive work has been undertaken to explain the transcriptional regulation of IkBα by MMP-12 in counteracting the protective anti-viral immune role of interferon-alpha (IFN α) [172,173].

### 6.2. Role in Cancer

Studies have shown that MMP-12 is involved in chronic obstructive pulmonary disease (COPD), emphysema, asthma, and arthritis [174]. Abdool et al. showed that MMP-12 degrades elastin in the lungs of smokers; it functions as a chemokine to enroll a pro-inflammatory immune response [4]. Moreover, MMP-12 is an identified mediator of arterial stiffening in both acute and chronic situations via the elastolytic effect of MMP-12 [4]. As a result, it may be involved in some vascular and neurological diseases such as atherosclerosis and aneurysms, spinal cord injury (SCI), multiple sclerosis (MS), Theiler murine encephalomyelitis, intracerebral haemorrhage (ICH), and ischemic stroke [19,175,176].

Additionally, MMP-12 is also implicated in the pathogenesis of different types of cancer; however, the risk of cancer susceptibility remains controversial [177,178]. Studies have documented that MMP-12 has antitumor activity against specific types of cancer, such as ovarian cancer [179] and colorectal cancer [180,181]. In contrast, researchers have reported that overexpression and functional polymorphism of MMP-12 is correlated with the occurrence and progression of colon cancer [182] and ovarian cancer [183]. Also, MMP-12 overexpression is a negative prognostic factor of hepatocellular carcinoma [184] breast cancer [185,186], oesophageal adenocarcinoma [187,188], skin cancer [189], and pancreatic cancer [186].

In addition, MMP-12 polymorphism relates to a higher risk of lung cancer dissemination, which has been documented by several studies [190,191,192]. Functionally, a defect in myeloid cells due to MMP-12 overexpression leads to abnormal myelopoiesis, immune suppression, and eventually the development of lung adenocarcinoma [193]. Furthermore, a meta-analysis that aimed to study the association between MMP-12 polymorphism and cancer concluded that the G allele of the MMP-12 82 A/G polymorphism may significantly increase the risk of epithelial ovarian cancer [177].

These studies confirm that MMP12 may be a significant regulator in the occurrence and growth of cancers.

### 6.3. Structure of Catalytic Domain

Structurally, MMP-12 is closely related to other MMPs, and it shares 49% sequence similarity with MMP-3 and MMP-1 [160]. MMP-12 is classified as having a medium-sized S1′ pocket that is characterized mostly by its hydrophobicity compared to other MMPs’ loop regions [17,194]. This hydrophobic environment is due to the presence of a series of residues (i.e., Ala234, Val235, Phe237, Lys241, Val243, and Phe248). Morales et al. have demonstrated the importance of hydrophobic interactions between S1′ pockets’ residues and inhibitors by developing a crystal structure of MMP-12 catalytic domain complex with non-zinc chelating inhibitors [194].

### 6.4. MMP-12 Inhibitors

Provided that MMP-12 is implicated in the development of a range of diseases, MMP-12 inhibitors are of interest in numerous areas of clinical therapy [19]. The number of MMP-12 inhibitors developed during the past decade is an indication of the significance of MMP-12 as a fundamental target in these diseases [19,195,196,197,198,199]. However, there are, to date, no MM-12 inhibitors documented in the literature that have effectively completed pre-clinical or clinical studies as a cancer therapy. As explained earlier, controlling the main central approaches of MMP-12 levels and activity have been pursued to develop compounds against MMP-12 activity [18]. Through the application of these strategies, various inhibitors of MMP-12 enzymatic activity have been developed in several diseases, though not in malignancies [22,200,201,202,203].

However, due to the extremely similar structures of the MMP family, nonselective MMP-12 inhibitors may interact with other MMPs, resulting in side effects [29]. Hence, the application of the rationale design of MMP-12 inhibitors would have a great effect on minimizing these undesirable effects by enhancing the selectivity of MMP-12 inhibitors. The rational design of selective MMP-12 inhibitors, based on the structure and specificity of MMP-12, comprises the introduction of a zinc-binding group (ZBG) to chelate the active site Zn(II) ion, an H-bond receptor or donor to interact with the amino acid backbone through hydrogen bonding, and a hydrophobic framework to fit into the S1′ pocket via hydrophobic interactions [17].

## 7. Matrix Metalloprotease-14 (MMP-14)

### 7.1. Function and Localization

MMP-14, previously known as MT1-MMP [204], was identified in 1994 and is classified as a membrane-type metalloproteinase [11]. MMP-14 is a protein that has been extensively studied and found in many different types of cells [205]. It plays an important role in various processes in our bodies. For example, it works on collagen I, as well as collagen II and III to some degree [206]. Also, it helps in the activation of the pro-MMP-2 protein, which is involved in cancer cell invasion [205].

Furthermore, MMP-14 works by forming a pair with itself on the cell surface and with MMP-2 and TIMP-2 to activate MMP-2 [207]. In addition to this, it is responsible for activating MMP-8 and -13 [204]. Moreover, MMP-14 has an important role in skeletal development, wound healing, inflammation, and many other functions [205]. Interesting to note is that MMP-14 can promote cell migration and invasion by breaking down the surrounding matrix [205]. Additionally, it acts as an enzyme that cleaves CD44, a hyaluronan receptor [207]. This cleavage enhances the growth and motility of cells through different mechanisms [205]. Beyond its enzymatic activity, MMP-14 interacts with different proteins and regulates processes like the immune response, bone development, and cell fusion [205]. MMP-14 is positioned at the forefront of moving cells, specifically on chromosome 14q11-q12 [62,204,208], and it is expressed at the cell surface [205].

### 7.2. Role in Cancer

In cases of human cancer, there is an overproduction and activation of MMP-14, which has been associated with the invasion and metastasis of cancer cells [209]. It plays a crucial role in enabling endothelial cells to invade and break down the surrounding tissue, ultimately facilitating the creation of new blood vessels [205]. This process is important in the development and progression of cancer [210,211]. The presence of MMP-14 has been linked to unfavorable outcomes in patients suffering from a variety of cancers [212], including melanoma [213]; advanced neuroblastoma [214]; mesothelioma [215]; lung cancer [216]; tongue, head, and neck carcinoma [217,218]; and bladder [219], breast [220], ovarian [221], pancreatic [222] and colorectal cancers [71,223]. Studies in animals have demonstrated that MMP-14 plays an important role in the spread of cancer cells to other parts of the body. Recent reviews have also suggested that MMP-14 can be used as an indicator of a patient’s prognosis for cancer [224].

### 7.3. Structure of the Catalytic Domain

The MMP-14 catalytic domain included an 8 amino acid sequence, PYAYIREG, that can affect the shape of the active site [204]. The S1′ pocket is shallow, since it contains leucine rather than arginine or tyrosine [1]. The hemopexin-like domain of MMP-14 plays a vital role in promoting the invasion of cells and enables it to interact with CD44. Also, it has a special site in its structure called a furin-like pro-protein convertase recognition site [1]. This site has a specific sequence of amino acids (RX[R/K]R) located at the end of the pro-domain of the protein. This special site allows for the protein to be activated through a process called proteolysis, in which the pro-domain is removed by specific enzymes [1]. Initially, the enzyme is produced as an inactive form, and it later becomes activated, removing the pro-peptide [225]. During secretion, the activation process takes place, and the activated enzyme is expressed on the cell surface [226].

### 7.4. MMP-14 Inhibitors

Computational chemistry techniques have been used to design and synthesize MMP-14 inhibitors for cancer treatment. These inhibitors work by targeting MMP-14 and preventing its ability to cleave substrates, which could potentially slow down or halt cancer progression. Virtual screening methods were also used to develop MMP-14 inhibitors [227]. Another way to discover inhibitors of MMP-14 is the high-throughput screening method, which involves testing large numbers of small molecules as potential MMP-14 inhibitors [228].

Researchers in the last decade have developed several small molecules of MMP-14 inhibitors (Table 6). Nuti and his team worked on improving the inhibitory activity of some compounds towards MMP-2, -9, and -14. They designed and tested a new series of N-isopropoxy-arylsulfonamide hydroxamates compounds. Of these, compound **31** was further analyzed for its binding mode to MMP-9 and-14 using X-ray crystallographic and docking studies [229]. Also, Cuffaro et al. tried to develop a dimeric compound (compound **32**) to reduce MMP-14-dependent activity on pro-MMP-2 activation, collagen degradation, and collagen invasion [230]. Their results showed that compound **32** had better dose-dependent results than its monomeric counterpart (compound **1**). Also, a virtual screening method was used to identify compound **33**, which is a hemopexin-targeting small molecule (NSC405020) [227]. In addition, Sijoli and his coworkers docked eight PAC-1 (compound **34**) and five isatin derivatives (compound **35**) into MMP-9 and -14. Then, they synthesized, purified, and tested these compounds to see whether they could inhibit the activity of MMP-9 and -14. Though the compounds did bind to the active sites of the enzymes, they were not strong inhibitors. Even when tested at high concentrations, the compounds did not significantly affect the activity of the enzymes [231]. Scientists at the University of Notre Dame Du Lac have developed special inhibitors for MMP-2, -9, and -14, which are involved in various neurological disorders. These inhibitors are water-soluble and can cross the blood–brain barrier to reach therapeutic concentrations in the brain. They are also designed to be cleared without causing harmful side effects in the central nervous system. One of the inhibitors (compound **36**), called ND-336, can inhibit all three enzymes simultaneously. The researchers believe that ND-336 and related compounds could be used to treat cancer [11].

## 8. Conclusions and Perspectives

Matrix metalloproteinases have been linked to a wide variety of pathological states including carcinogenesis. The association between different members of the MMP family and cancer suggests that their inhibition could profoundly impact the suppression of tumor growth and metastasis. The selective inhibition of targeted matrix metalloproteinases remains a hurdle in successfully developing MMP inhibitors for clinical trials, despite the availability of structural data. In this review, we have reported the various approaches used in the design and generation of MMP (1, 2, 3, 7, 12, and 14) inhibitors accompanied by their anticancer activities. The comprehensive nature of the review emphasizes the growing interest in targeting MMPs for anticancer drug development and may shed light on multiple avenues to develop inhibitors that specifically target different MMPs implicated in cancer progression.

## Figures and Tables

**Table 1:** Classification of the main members of the MMP family and their group of substrates [10].

Traditional Classification (Common Name)	Numerical Classification	Group of Substrates
*Collagenases*		
Collagenase-1	MMP-1	Collagen (I, II, III, VII, VIII, X), casein, entactin, laminin, pro-MMP-1, -2, -9, and serpins
Collagenase-2	MMP-8	Collagen (I–III, V, VII, VIII, X), gelatin, aggrecan, fibronectin
Collagenase-3	MMP-13	Gelatin, collagen (IV–VI, X), elastin, fibronectin
*Gelatinases*		
Gelatinase A	MMP-2	Gelatin, collagens (IV, V, VII, X, XIV), elastin, fibrillin, osteonectin
Gelatinase B	MMP-9	Gelatin, collagens (IV, V, VII, X, XIV), elastin, fibrillin, osteonectin
*Stromelysins*		
Stromelysin-1	MMP-3	Laminin, aggrecan, gelatin, fibronectin
Stromelysin-2	MMP-10	Collagens (III–V), gelatin, casein, aggrecan, elastin, MMP-1,8
Stromelysin-3	MMP-11	Fibronectin, laminin, aggrecan, gelatin
*Matrilysins*		
Matrilysin-1	MMP-7	Collagen (IV–X), fibronectin, laminin, gelatin, aggrecan, pro-MMP-9
Matrilysin-2	MMP-26	Gelatin, collagen IV, pro-MMP-9
*Macrophage Metalloelastase*		
Macrophage Metalloelastase	MMP-12	Elastin, gelatin, collagen I, IV, fibronectin, laminin, vitronectin, proteoglycan
*Membrane-type MMPs (MT-MM)*		
MT-MMP-1	MMP-14	Collagen (I, II, III), gelatin, fibronectin, laminin aggrecan, tenascin
MT-MMP-2	MMP-15	Fibronectin, laminin, aggrecan, perlecan
MT-MMP-3	MMP-16	Collagen III, gelatin, casein
MT-MMP-4	MMP-17	Fibrinogen, TNF precursor
MT-MMP-5	MMP-24	Proteoglycans

**Table 2:** Chemical structures and potency of synthetic MMP-1 inhibitors.

Compound	Potency	Chemical Structure	Reference
**1**	IC_50_ = 0.4 µM	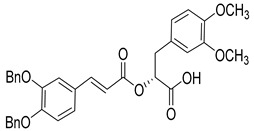	[56]
**2**	IC_50_ = 0.034 µM	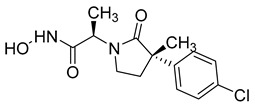	[57]
**3**	IC_50_ = 20 µg/mL (MCF-7)	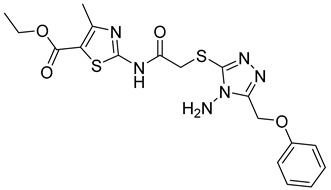	[58]
**4**	Ki = 77 nM	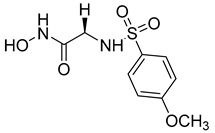	[59]
**5**	IC_50_ = 0.18 µM	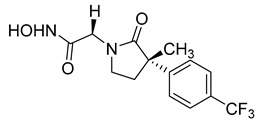	[60]

**Table 4:** Chemical structures and potencies of synthetic MMP-3 inhibitors.

Compound	Potency	Chemical Structure	Reference
**24**	Ki = 43 nM	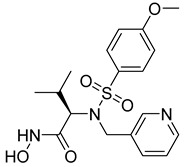	[38]
**25**	Ki = 19 nM	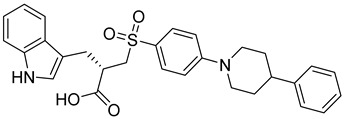	[38]
**26**	Ki = 15 nM	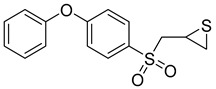	[38]
**27**	IC_50_ = 1 nM	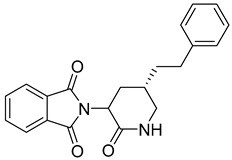	[128]
**28**	IC_50_ = 0.4 µM	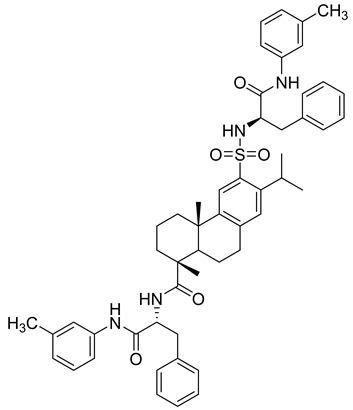	[129]

**Table 5:** Chemical structures and potencies of synthetic MMP-7 inhibitors.

Compound	Potency	Chemical Structure	Reference
**29**	Ki = 3.7 µM	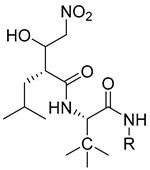	[159]
**30**	IC_50_ = 2.2 µM	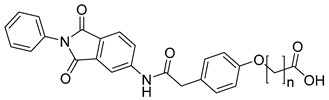	[158]

**Table 6:** Chemical structures and potencies of synthetic MMP-14 inhibitors.

Compound	Potency	Chemical Structure	Reference
**31**	IC_50_ = 3.9 nM	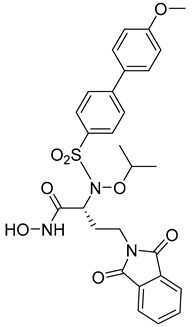	[229]
**32**	IC_50_ = 14 nM	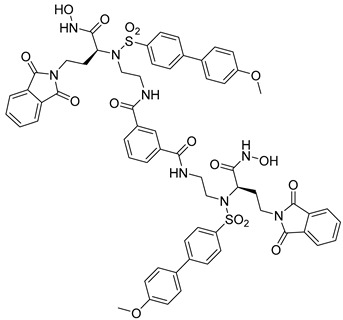	[230]
**33**	IC_50_ > 100 μM	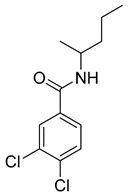	[227]
**34**	-	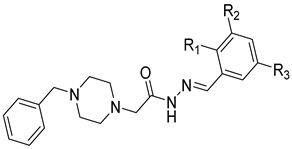	[231]
**35**	-	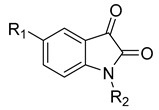	[231]
**36**	Ki = 0.120 µM	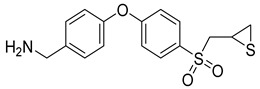	[11]

## Data Availability

Data supporting the findings of this study are available within the article.
